# Chronic features of allergic asthma are enhanced in the absence of resistin-like molecule-beta

**DOI:** 10.1038/s41598-018-25321-y

**Published:** 2018-05-04

**Authors:** Kim S. LeMessurier, Maneesha Palipane, Meenakshi Tiwary, Brian Gavin, Amali E. Samarasinghe

**Affiliations:** 1 Department of Paediatrics, University of Tennessee Health Science Center, Memphis, TN 38103 USA; 2 Children’s Foundation Research Institute, Memphis, TN 38103 USA

## Abstract

Asthma is characterized by inflammation and architectural changes in the lungs. A number of immune cells and mediators are recognized as initiators of asthma, although therapeutics based on these are not always effective. The multifaceted nature of this syndrome necessitate continued exploration of immunomodulators that may play a role in pathogenesis. We investigated the role of resistin-like molecule-beta (RELM-β), a gut antibacterial, in the development and pathogenesis of *Aspergillus*-induced allergic airways disease. Age and gender matched C57BL/6J and *Retnlb*^−/−^ mice rendered allergic to *Aspergillus fumigatus* were used to measure canonical markers of allergic asthma at early and late time points. Inflammatory cells in airways were similar, although *Retnlb*^−/−^ mice had reduced tissue inflammation. The absence of RELM-β elevated serum IgA and pro-inflammatory cytokines in the lungs at homeostasis. Markers of chronic disease including goblet cell numbers, *Muc* genes, airway wall remodelling, and hyperresponsiveness were greater in the absence RELM-β. Specific inflammatory mediators important in antimicrobial defence in allergic asthma were also increased in the absence of RELM-β. These data suggest that while characteristics of allergic asthma develop in the absence of RELM-β, that RELM-β may reduce the development of chronic markers of allergic airways disease.

## Introduction

Cellular and molecular composition of the respiratory tract is vast with complicated interplay required for homeostasis. Often times, structural cells and their mediators are altered in response to encounters with innocuous or pathogenic agents leading to dysregulation. Allergen-induced structural and functional changes in airways and parenchyma, while initiated by the bronchial epithelia^1^, are perpetuated by a misguided immune response^2^. Allergic asthma is a syndrome that impacts over 235 million people globally^3^ and approximately 8% of the population in the United States^4^. Generally considered as an inflammatory disorder with a T_H_2 bias, structural changes including goblet cell (GC) metaplasia and airway wall remodelling events, contribute to physiologic dysfunction in breathing that can require medical attention.

Airway structural cell- and immune system-derived mediators contribute to asthma pathogenesis. Resistin-like molecules (RELMs) are small (105–114 amino acids) secreted proteins characterized by conserved cysteine rich carboxyl domains^5^. Family members, RELM-α, RELM-β, resistin, and RELM-γ, found in the gut, lungs, and adipose tissue^6^, have known functions in the pathophysiology of metabolic^7^ and inflammatory diseases^8^. While mice express the entire spectrum of RELM proteins, only resistin and RELM-β, which share sequence homology, have been identified in humans to date^5^. RELM-β was identified and characterized in the gut^5,9,10^, but also has ascribed functions in the lung, including participation in inflammation, GC hyperplasia, and fibrosis^11,12^. RELM-β expression is prominent in alveolar epithelial cells, and its known functions in lung inflammation and proliferation of epithelial cells and fibroblasts have led to its categorization as a marker of allergic asthma in ovalbumin and bleomycin mouse models of asthma and fibrosis^11–13^. Limitations in mouse models such as rapid reversal of inflammation and lack of remodelling (in ovalbumin models), and excessive inflammation and reversible fibrosis (in bleomycin models)^14,15^, necessitate the characterization of RELM-β in a robust chronic model of respiratory allergy that utilizes clinically relevant allergen exposures.

Understanding the functions of RELM proteins in the context of the lung may be important in delineating previously unknown mechanisms that drive pulmonary diseases, including asthma. Most asthmatics without co-morbidities or respiratory infections have elevated RELM-β in the bronchoalveolar lavage (BAL) fluid and in bronchial biopsies compared to controls^16^. We noted elevated RELM-β in the lungs of mice in our model of asthma and influenza (unpublished), which have an altered immune phenotype^17,18^. Therefore, we hypothesized that RELM-β regulates the development of features of severe asthma with fungal sensitization (SAFS) in response to clinically relevant *Aspergillus fumigatus* fungal antigens. Our data contradict previous reports that RELM-β promotes macrophagic inflammation, GC metaplasia, and subepithelial fibrosis^11–13^, and instead, suggest a function for RELM-β as a negative regulator of chronic features in allergen-associated lung disease.

## Results

**Figure 1:**
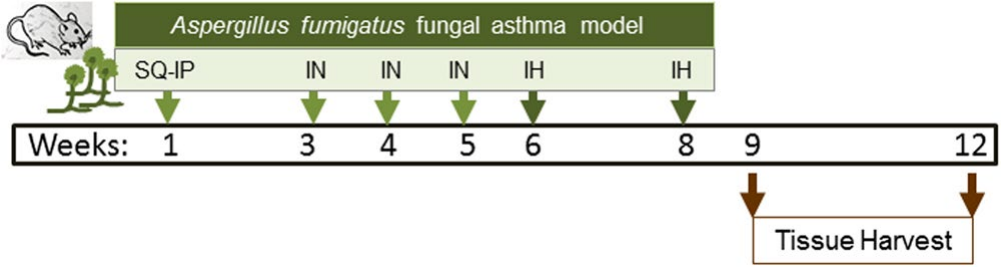
Schematic representation of the timeline in the severe asthma with fungal sensitization model (SAFS). Age and gender-matched mice are administered antigen from *Aspergillus fumigatus* extract via subcutaneous (SQ) and intraperitoneal (IP) injections. Following a 2 week rest period, antigen is administered via intranasal (IN) route once a week for three weeks. Sensitized mice are then exposed to conidia liberated from live fungal cultures for 10 minutes at a time two weeks apart via inhalation (IH). Samples were harvested at days 7 and 35 after the second fungal challenge.

Characteristics of allergic asthma include peribronchovascular (PBV) inflammation, GC metaplasia, and airway wall remodelling events in addition to physiologic changes leading to increased airway hyperresponsiveness (AHR) and an IgE-biased humoral immune response. Pathways that influence the development and pathogenesis of allergic asthma have been elucidated using mouse models, and those that can recapitulate the acute and chronic features of asthma are especially important to delineate functions of cells and their mediators in disease. Herein, using our mouse model of SAFS (Fig. 1), we investigated the role played by RELM-β in the development of characteristics associated with SAFS.

### RELM-β minimally impacts allergic inflammation

**Figure 2:**
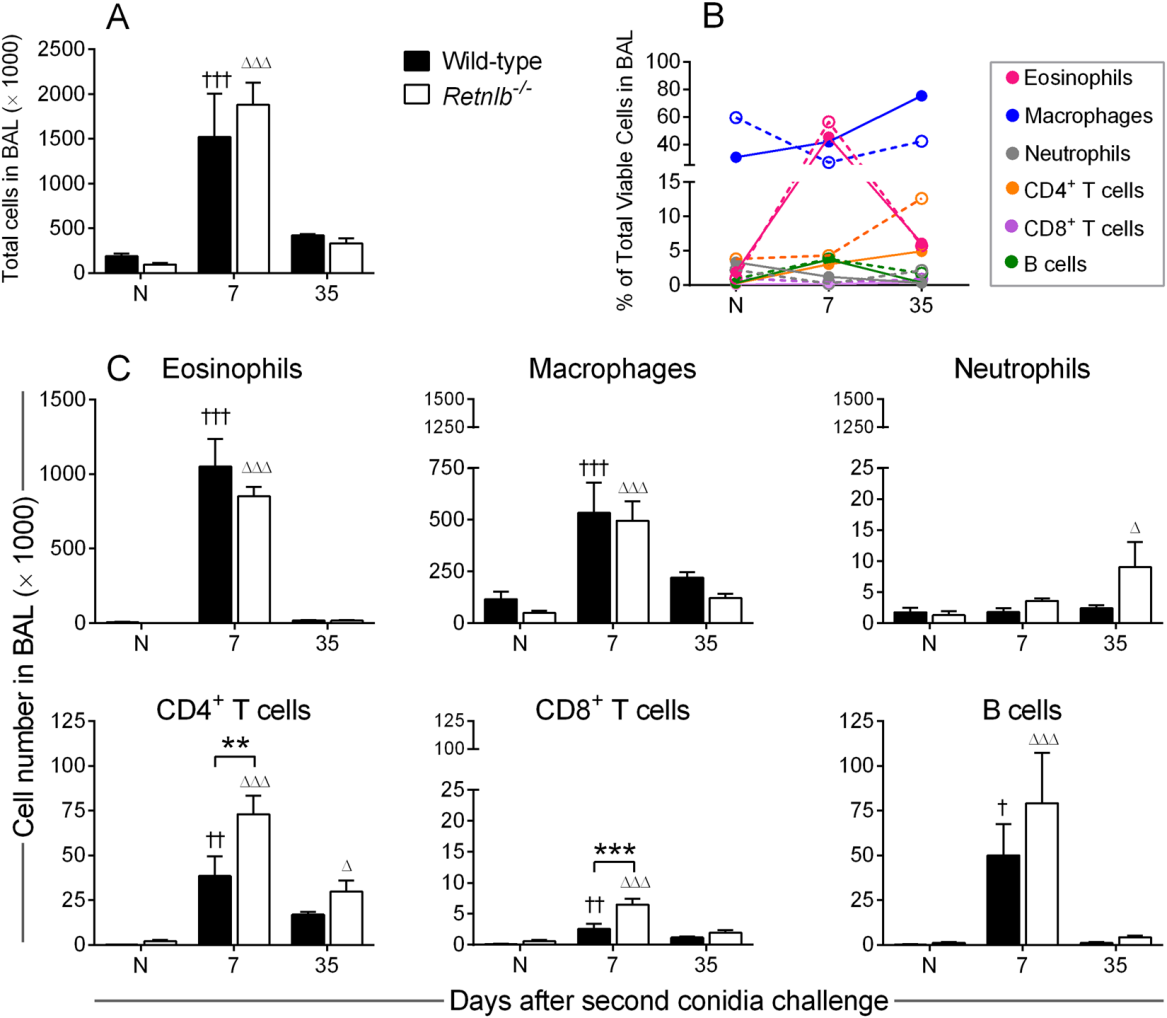
RELM-β has a minimal impact on airway inflammation. Immune cells were recruited into the airways (collected by bronchoalveolar lavage) in both *Retnlb* sufficient (wild-type) and deficient (KO) mice (**A**). Cell populations normalized to cell number showed slight variations in the kinetics between WT (solid circles) and KO (open circles) mice (**B**). Absolute cell numbers suggest no major differences in innate cell populations at early the early time point although neutrophils were present at the late time point in KO airways (**C**). RELM-β deficient mice had more T cells, while B cell numbers were similar (**C**). Data are represented as the mean and SD of n = 4–7 mice/group in one representative study of two independent studies. Data at each time point were compared by two-way ANOVA with Dunnett’s and Sidak’s multiple comparisons test to their naïve controls († and Δ), and between genotypes (*) respectively. One, two, and three symbols represent P < 0.05, P < 0.01, and P < 0.001 correspondingly. N – Naïve.

**Figure 3:**
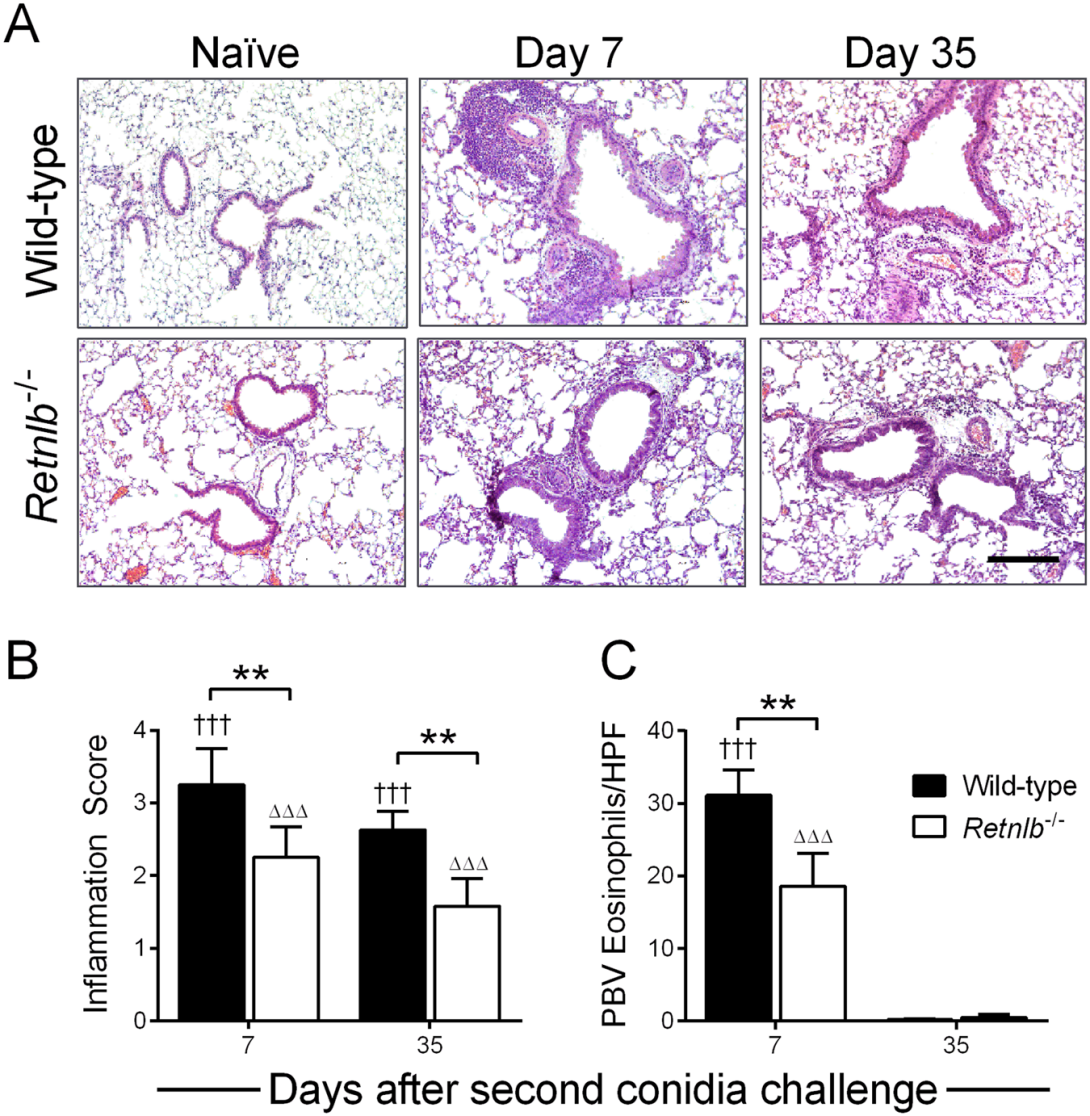
RELM-β promotes peribronchovascular inflammation. Peribronchovascular inflammation was highest at early time points and gradually decreased over time in both genotypes compared to naïve controls (**A**), and *Retnlb*^−/−^ mice had less tissue inflammation compared to WT (**B**). Tissue eosinophils were lower in KO mice compared to WT (**C**). Scale bar = 200 µm and applicable to all photo micrographs. Naïve mice in each genotype were scored 0 and did not contain tissue eosinophils. Data are represented as the mean and SD of n = 4–7 mice/group in one representative study of two independent studies. Data at each time point were compared by two-way ANOVA with Dunnett’s and Sidak’s multiple comparisons test to their naïve controls († and Δ), and between genotypes (*) respectively. One, two, and three symbols represent P < 0.05, P < 0.01, and P < 0.001 correspondingly. HPF – high power field.

Inflammation is temporally regulated after allergen exposure with waves of infiltrating cells that belong to both innate and adaptive branches of the immune system. BAL was performed, and harvested leukocytes were enumerated and profiled by flow cytometry (Fig. 2) while tissue inflammation was qualitatively determined by histologic analyses (Fig. 3). The total cell population in both WT and *Retnlb*^−/−^ mice increased after allergen challenge and gradually depleted over time with no major differences between the genotypes (Fig. 2A). Slight differences in cell kinetics were noted when data were normalized to total cell numbers in each group (Fig. 2B). Eosinophils in the airways were similar between the WT and KO mice (Fig. 2B). While macrophage numbers were similar between the genotypes (Fig. 2C), when taken as a percentage of total cells, KO mice had more macrophages at baseline which reduced after allergen challenge. (Fig. 2B). This model of SAFS has minimal neutrophil influx due to eosinophilic dominance^19^. However, neutrophils were found in the airways of *Retnlb*^−/−^ mice at the late time point (Fig. 2C). Early recruitment of CD4^+^ and CD8^+^ T cells occurred in the absence of RELM-β (Fig. 2C) and KO mice had a larger percentage of CD4^+^ T cells in the airways at the late timepoint (Fig. 2B).

Tissue inflammation can persist for long periods of time after allergen exposure and considered to participate in airway physiologic responses in asthma^20^. PBV inflammation was apparent in both genotypes after allergen challenge compared to baseline (Fig. 3). Tissue inflammation was scored through a blinded process and the areas of inflammatory foci were reduced in the *Retnlb*^−/−^ mice compared to WT controls (Fig. 3B). The number of eosinophils in the PBV areas was reduced in KO mice by one week after allergen challenge (Fig. 3C). While airway inflammation was largely resolved by Day 21 in both genotypes (data not shown), PBV inflammation was diminished but not fully resolved at the final time point tested (Figs 2 and 3). Cumulatively, these data suggest that the absence of RELM-β may only have a minimal impact on allergic inflammation.

### RELM-β may inhibit goblet cell metaplasia

**Figure 4:**
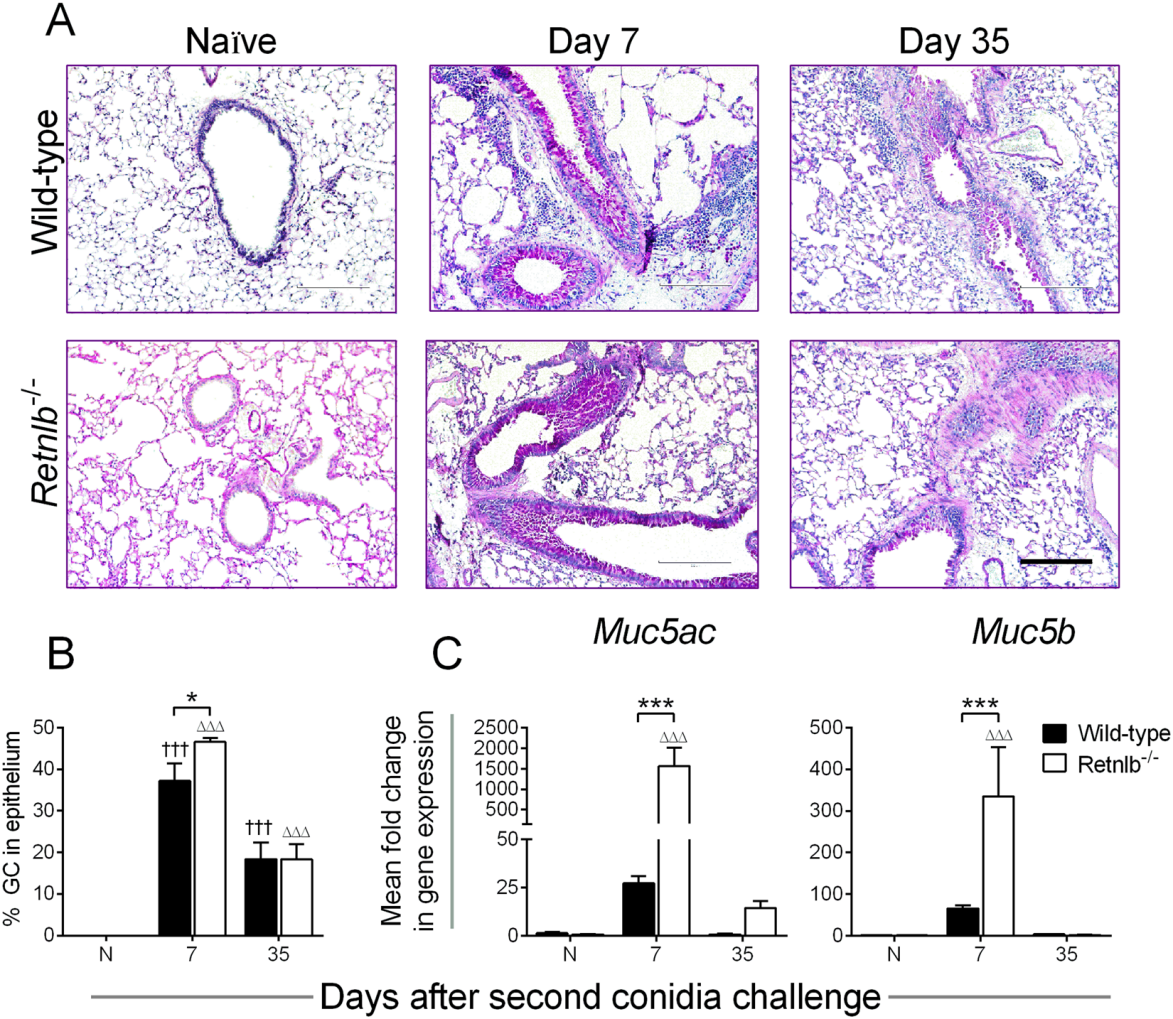
RELM-β inhibits goblet cell (GC) differentiation and *Muc* gene expression. Goblet cells were not observed in the naïve (N) controls of either genotype but were abundant in the airway lining after allergen exposure (**A**). While GCs consisted of approximately 40% of the epithelial lining in both genotypes at the early time point, these cells reduced over time to only about 20% at the late time point (**B**). More GCs were present in the airways of *Retnlb*^−/−^ mice at Day 7 (**B**), corresponding with greater expression of mucus genes, *Muc5ac* and *Muc5b* (**C**). Scale bar = 200 µm applicable to all photo micrographs. Data are represented as the mean and SEM of n = 4–7 mice/group in one representative study of two independent studies. Data at each time point were compared by two-way ANOVA with Dunnett’s and Sidak’s multiple comparisons test to their naïve controls († and Δ), and between genotypes (*) respectively. Three symbols represent P < 0.05 and P < 0.001 correspondingly.

Increased mucus producing cells and mucus hypersecretion are hallmarks of airway inflammation-associated diseases such as allergic asthma. GCs are the primary producers of RELM-β in the gut^21^ where it is known to be important for anti-parasitic host defence^22^, barrier integrity^10^, inflammation^23,24^, and microbiome maintenance^25,26^. We investigated GC numbers and mucin genes in both genotypes expecting to find them reduced in the KO mice based on previous reports^11^. GCs lining the airways were visualized by periodic acid Schiff’s stain (Fig. 4A) and enumerated (Fig. 4B). GCs were elevated early after allergen challenge in both genotypes, but KO mice had noticeably more GCs lining the airways compared to WT, although this trend was not observed at the late time point (Fig. 4B). Corresponding expressions of mucin genes considered as markers of GC metaplasia^27^, *Muc5ac* and *Muc5b*, were markedly elevated in KO mice at early time points (Fig. 4C).

### Airway wall remodelling is increased in the absence of RELM-β

**Figure 5:**
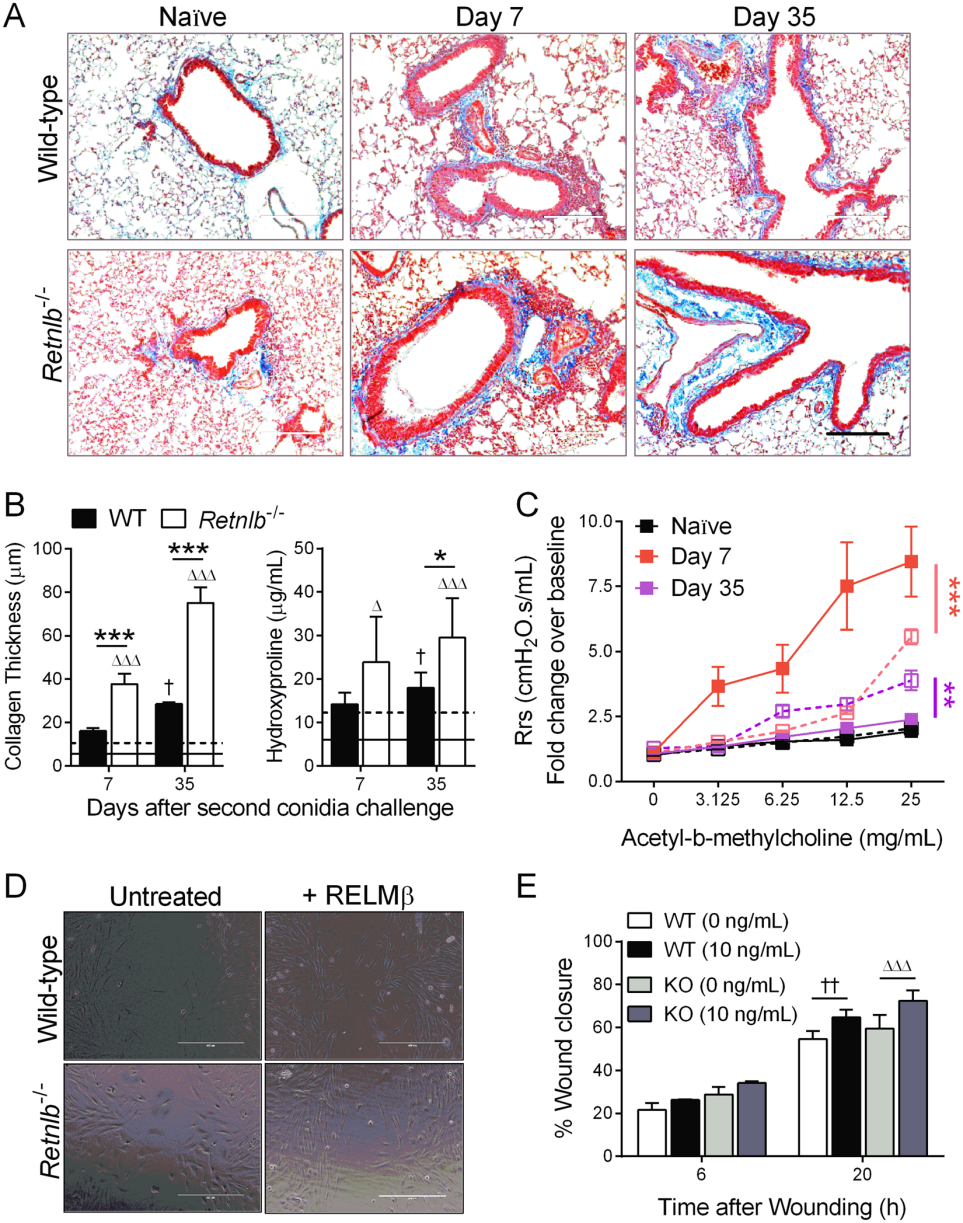
Airway wall remodelling may impact physiologic responses in the absence of RELM-β. Airway wall remodelling was observed qualitatively with trichrome staining (**A**) wherein *Relnlb* KO mice have increased peribronchovascular collagen deposition and hydroxyproline compared to wild-type (WT) controls (**B**). Resistance of the respiratory system (Rrs) in response to nebulized methacholine was significantly higher in the WT (closed squares) early after allergen challenge, but this increase persisted in the KO (open squares) at the late time point (**C**). Primary lung fibroblasts isolated from WT and KO mice were wounded and treated/not with 10 ng/mL recombinant RELM-β protein. Wound closure was measured at varying time points based on the initial width of the wound in each replicate, and a significant difference was noted at 20 hours in fibroblasts from both genotypes (**D**,**E**). Lines in B represent naïve WT (solid) and KO (dotted). Scale bar = 200 µm (applicable to all in **A**) and 400 µm (applicable to all in **D**). Data are represented as the mean and SEM of one representative study with n = 4–7 mice/group (**A**–**C**) and as mean and SD of n = 3 wells/group (D-E). Data were analysed by two-way ANOVA with Tukey’s multiple comparisons test. Two and three symbols represent P < 0.01, and P < 0.001 correspondingly. N – Naïve.

Airway wall remodelling is a characteristic of severe and chronic asthma that can be irreversible. We observed subepithelial fibrosis as a feature of remodelling with trichrome staining (Fig. 5A) and noted that KO mice had more collagen deposition than their WT counterparts especially at late time points (Fig. 5B). Hydroxyproline levels were also elevated in KO mouse lungs compared to WT (Fig. 5B). AHR was measured after methacholine challenge as a physical attribute of inflammation and stiffness of the airways. While airway resistance was higher in WT mice at the early time point, KO mice had elevated responses throughout the time course (data not shown) which remained high at Day 35 (Fig. 5C). Fibroblasts play architecturally important functions in the lungs by providing structural support through matrix deposition, but, can also contribute to lung pathology through cytokine production^28^ and differentiation into myofibroblasts^29^. Fibroblast migration and proliferation are initial steps in fibrotic remodelling in allergic asthma^30^. In order to determine if elevated collagen in the absence of RELM-β was due to altered fibroblast growth/migration/expansion during disease, we measured closure of a scratch wound in the presence/absence of recombinant RELM-β in primary lung fibroblasts from WT and KO mice (Fig. 5D). Wound closure was calculated as a decrease in wound size at each time point. Fibroblasts from KO mice were responsive to exogenous RELM-β, but, our data suggest that while RELM-β may be beneficial for fibroblast outgrowth, it was not necessary (Fig. 5E).

### RELM-β deficiency affects humoral immune responses to allergen challenge

**Figure 6:**
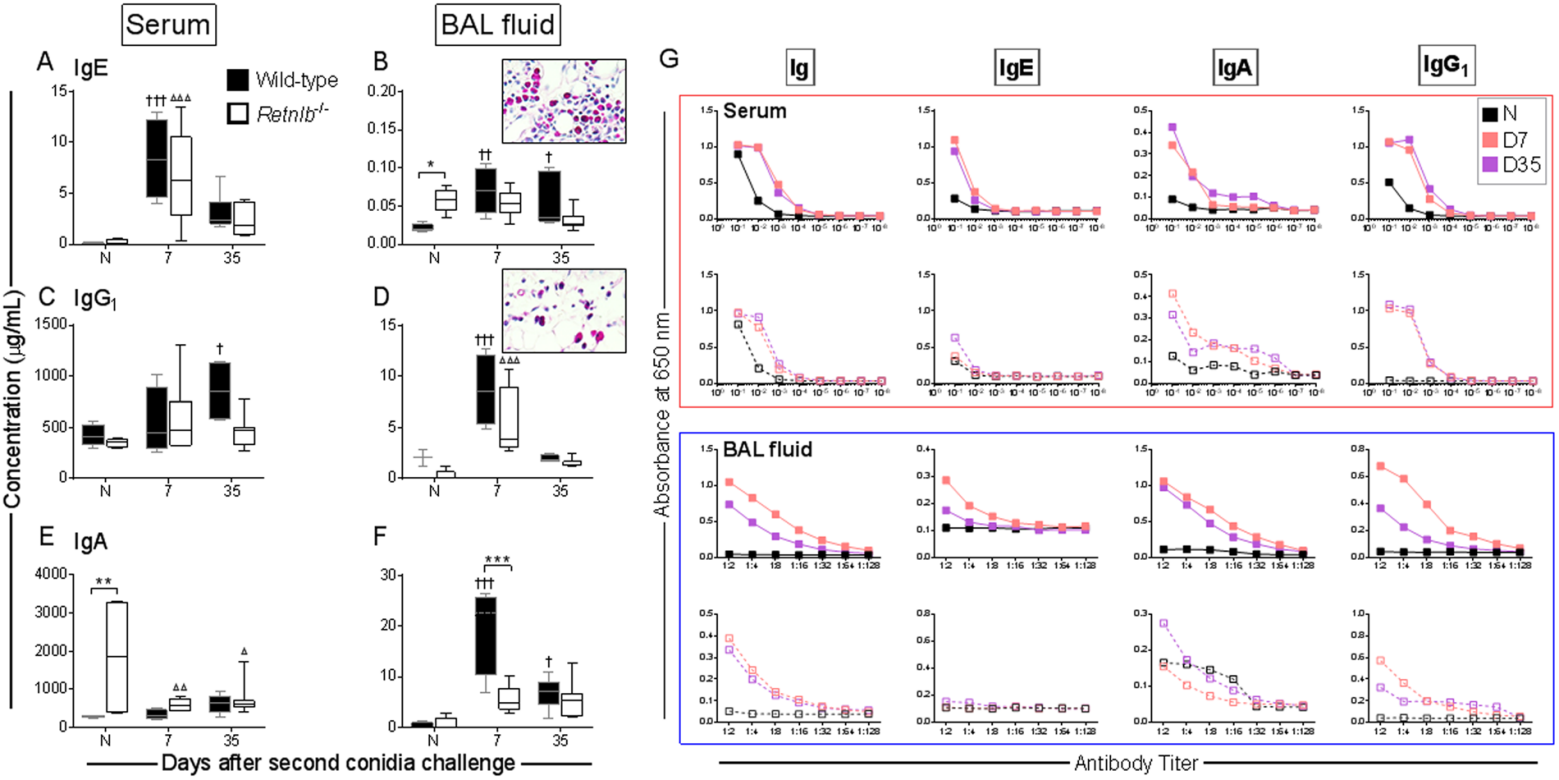
Systemic and local antibody responses were induced in both wild-type (WT) and *Retnlb* null mice (KO). Antibody levels in the serum and bronchoalveolar lavage (BAL) fluid were measured at each time point after the second fungal challenge. Allergen exposure led to an increase in immunoglobulin (Ig) E levels in the serum (**A**). IgE in the BAL fluid also increased after allergen exposure in WT mice, but did not change over baseline in the KO group which had significantly more IgE at baseline, compared to WT (**B**). Serum IgG_1_ levels increased after allergen challenge but did not reach significance until the late time point in the WT mice (**C**). Changes in the mucosal IgG_1_ was apparent at early time points in both groups (**D**). Steady state systemic IgA was high in the KO mice, which reduced after allergen challenge while WT IgA in the serum increased minimally over naïve controls (**E**). IgA levels in the BAL fluid increased in response to allergen provocation in the WT and this increase was delayed in the KO, but did not reach statistical significance (**F**). More Mott cells (magenta coloured) were noted in the WT lungs (inset **B**) than in KO (inset **D**). *Aspergillus fumigatus*-specific antibodies were produced in both groups but generally more abundant in the WT mice compared to KO (**G**). Data are represented as the mean and SD of n = 4–7 mice/group in one representative study of two independent studies. Data shown as range from minimum to maximum where lines and dots represent the median and mean respectively. Data analysed with two-way ANOVA with Sidak’s and Dunnett’s multiple comparisons test to compare data between groups at each time point (*) or to naïve controls within groups († in WT and Δ in KO) respectively where significance values *p* < 0.05, *p* < 0.01, and *p* < 0.001 are denoted by one, two, or three symbols respectively. N – Naïve; D – Day.

Increased systemic IgE after allergen challenge marked the induction of allergy in both genotypes (Fig. 6A). Local IgE levels were higher in the *Retnlb*^−/−^ mice at baseline compared to WT controls and did not change as a result of allergen provocation (Fig. 6B). Systemic IgG_1_ was only increased over baseline at late time points in WT mice and remained equivalent to baseline in the KOs (Fig. 6C). In contrast, local IgG_1_ levels rapidly increased in both genotypes and returned to baseline levels by Day 35 post-allergen challenge (Fig. 6D). Systemic IgA in naïve *Retnlb*^−/−^ mice was highly elevated, although allergen challenge caused a rapid reduction (Fig. 6E). The increase in local IgA that occurred after allergen challenge in the WT mice, did not occur in the KO mice where IgA was maintained at a concentration similar to that in the naïve animals throughout the time course (Fig. 6F). We have previously found that antibody producing B cells are dynamically regulated in this model of SAFS^18,31^. Although B cells were not the focus of this investigation, we did note that there were more Mott cells in the WT lungs (inset Fig. 6B) compared to KO (inset Fig. 6D), which suggests that immunoglobulin production/producing cells may be directly/indirectly regulated by RELM-β. Titres of *A. fumigatus*-specific isotypes were measured to determine if a difference in specificity of antibodies occurred between the genotypes tested. *A. fumigatus*-specific immunoglobulin (Ig) titres were generally similar between the two genotypes (Fig. 6G). Although *Retnlb*^−/−^ mice had elevated serum IgE, the fungal-specific IgE titre in the sera was low in these mice and there was no *A. fumigatus*-specific IgE in the KO mouse BAL fluid (Fig. 6G).

### Mediators of allergy and immunity are dynamically impacted by the absence of RELM-β

**Figure 7:**
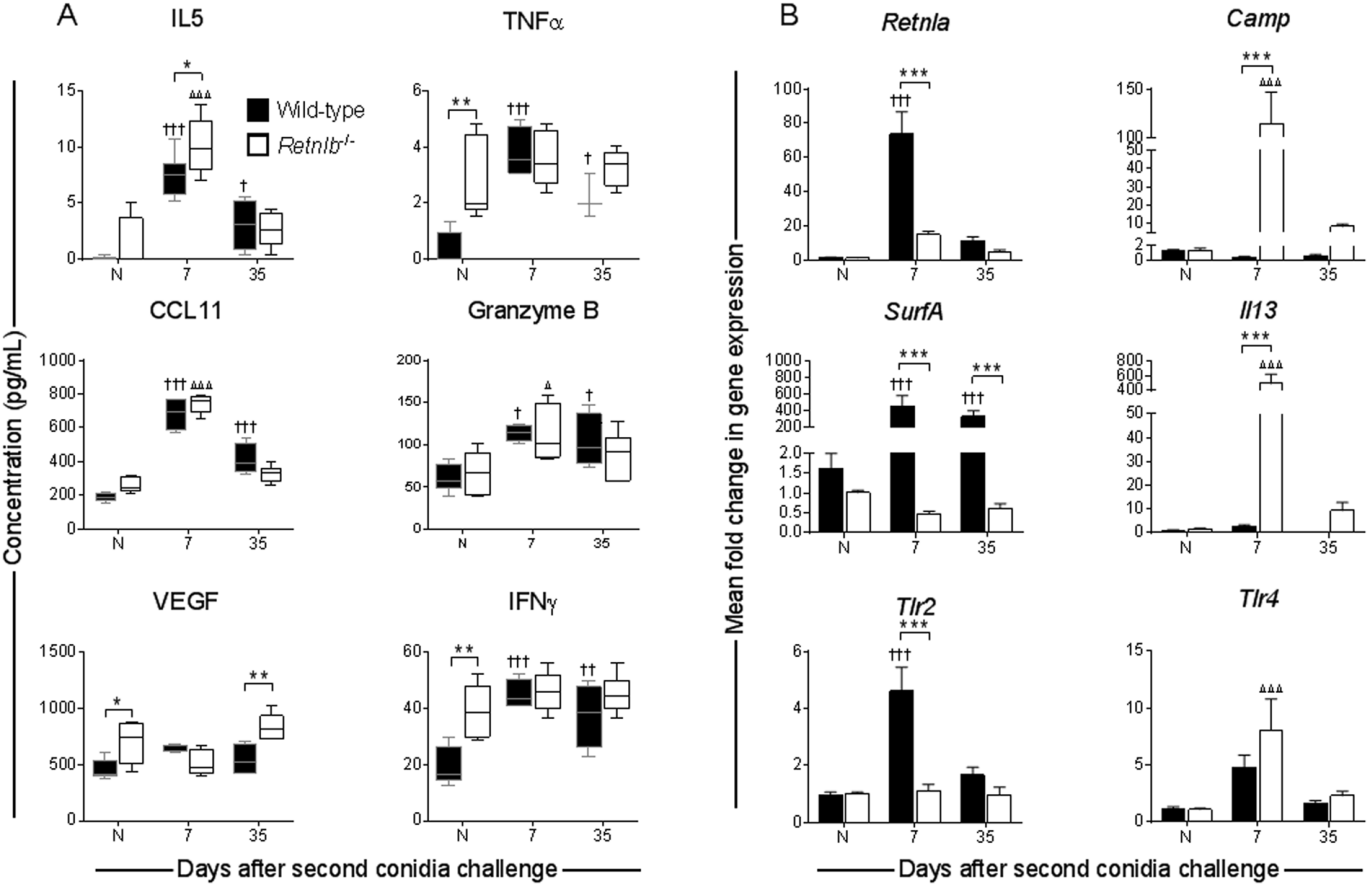
Biomarkers of inflammation increased in response to allergen challenge. Cytokines in the bronchoalveolar lavage fluid were measured in each genotype and found to be elevated in mice subjected to the fungal asthma model. Notably, *Retnlb* deficient mice had elevated TNFα, VEGF, and IFNγ at steady state compared to WT controls (**A**). Wild-type mice had rapid increase in cytokines at early time points with gradual depletion over time whereas *Retnlb* KO followed similar trends in cytokine production, albeit at slightly lower levels at early time points (**A**). Wild-type mice had markedly elevated expression of *Retnla* and *SurfA* in the lung while *Retnlb* null mice had markedly elevated *Camp* and *Il13* (**B**). Expression of *Tlr2* dominated at the early time point in the wild-type mice while *Trl4* took precedence in the *Retnlb* null mice (**B**). Data shown as range from minimum to maximum where lines and dots represent the median and mean respectively (**A**) and as the mean and SD (**B**) of n = 4–7 mice/group and representative of one independent study of two. Data analysed with two-way ANOVA with Sidak’s and Dunnett’s multiple comparisons test to compare data between groups at each time point (*) or to naïve controls within groups († in WT and Δ in KO) respectively, where significance values *p* < 0.05, *p* < 0.01, and *p* < 0.001 are denoted by one, two, or three symbols respectively. N – Naïve.

Since asthma is an inflammatory disease with multiple leukocyte involvement, cytokines and growth factors in the microenvironment are important to activate/deactivate both immune and structural cells during disease pathogenesis. Mediators that are of known importance to allergy and host defence in the lungs were selected for comparative analysis (Fig. 7). Interestingly, naïve *Retnlb*^−/−^ mice had elevated pro-inflammatory mediators, TNFα, VEGF, and IFNγ, compared to naïve WT controls (Fig. 7A). As expected, IL-5 and CCL11 were elevated soon after allergen exposure in both genotypes (Fig. 7A). Emphasizing the complexity of asthma, markers of T_H_1 responses, such as TNFα and IFNγ were also elevated in mice of both genotypes (Fig. 7A). IL-13 is a key T_H_2 cytokine known to have functions in AHR and airway remodelling, among others, during allergy. IL-13 promotes RELM-β in the gut^32^ possibly through enhancement of GC metaplasia and also induces RELM-β production by airway epithelial cells^16^. However, we found that *Il13* was highly expressed in mice deficient in *Retnlb* compared to WT controls (Fig. 7B).

Granzyme B, a serine protease secreted by various leukocytes including T cells^33^ and elevated in asthmatics^34^, has diverse functions including regaining homeostasis by killing T_H_2 cells^35^. Since we noted an increase in T cells in the BAL of KO mice (Fig. 2C), we quantified granzyme B as a marker of T cell activation in the airways and found that allergen exposure resulted in an increase in granzyme B levels in both genotypes (Fig. 7A). Vascular endothelial growth factor (VEGF), also known to be increased in asthmatics^36^, was increased in KO mice at baseline but not further induced by allergen exposure (Fig. 7A).

Additional mediators that rapidly turn over or are not secreted were measured as changes in gene expression. Differences in expression of selected genes between the two genotypes reached significance at early time points but were similar by the late time point (Fig. 7B). The expression of *Retnla* was measured to determine if there may be a compensatory mechanism in the KO mice. While *Retnla* was rapidly increased in WT mice after allergen challenge, its increase in the KO mice was comparatively less (Fig. 7B). Pulmonary surfactants line the airways and reduce the surface tension between the tissue and air, and surfactant A has been suggested to have protective and detrimental roles in asthma^37,38^. While WT mice had a significant increase in *SurfA* expression, this gene was not affected by allergen exposure in KO animals (Fig. 7B). Cathelicidin antimicrobial peptide (CAMP) is a bactericidal^39^ agent that activates eosinophils^40^, and is upregulated during virus infection^41^, but, its role in asthma is unclear. Since RELM-β also has antimicrobial functions, we investigated how its absence may impact *Camp* after allergen challenge and found that *Retnlb* KO mice had rapid upregulation of *Camp* which did not occur in the WT (Fig. 7B). Since our chosen allergen is known to regulate toll-like receptor (TLR)-responses to activate innate immune responses^42^, we investigated *Tlr2* and *Tlr4* expression in the lungs and found that WT mice had high expression of *Tlr2* while KO upregulated *Tlr4* (Fig. 7B).

## Discussion

Lack of understanding in disease aetiology, treatment insensitivity, and complications in the pathogenesis and genetic-environmental interactions are only some reasons for the delay in identifying a cure to asthma. While various mediators from immune, endocrine^43^, and neural systems^44^ have been demonstrated to function in the pathogenesis of allergic asthma, complex interactions between systems and their products decrease the likelihood that a single molecule can be targeted as the initiator/perpetuator of this condition. A number of studies have implicated RELM-β as a pro-fibrogenic mediator during airways disease^11–13,16^. We noted that RELM-β was dynamically regulated upon virus infection in our model of asthma and influenza morbidity (unpublished). Since its role had not been previously determined in a mouse model that utilizes a clinical allergen in its natural route of exposure, we investigated inflammatory and molecular profiles in *Retnlb*^−/−^ mice subjected to an *A. fumigatus*-induced model of SAFS to determine its role in pathogenesis. We report that the absence of RELM-β influenced inflammation, increased GC metaplasia, worsened subepithelial fibrosis, and sustained airway resistance. Based on these data, we suggest that RELM-β mitigates the development of characteristics associated with SAFS, which warrants further investigation into the mechanism by which RELM-β may influence features of chronicity in airways disease.

Airways inflammation, eosinophilia, and serum IgE are hallmarks of fungal asthma that can be modelled effectively in mice^19,45^. Activation of recruited inflammatory cells at the barrier can lead to hyperactivation of epithelia and fibroblasts^46^ via immune-epithelial cross-talk. Inflammation in this SAFS model may be initiated by TLR activation in bronchial epithelia leading to pro-inflammatory cytokine and cell recruitment that continues through positive feedback. Recombinant RELM-β activates gut macrophages^23^ and its administration into the airways leads to macrophage dominant inflammation in the airways^11^, suggesting a pro-inflammatory function for this molecule. Although allergen exposure resulted in an increase in airway macrophages, since there was no difference between the groups, RELM-β may not be important in macrophage recruitment or egression. However, when data were normalized to the cell numbers in the airways, we noted that naïve KO mice had more macrophages. Since airway macrophages are a source of RELM-β^13^, it would be of interest to determine the phenotypic and functional characteristics of these macrophages.

Eosinophils and T_H_ cells typically take centre stage in asthma studies. Eosinophils in the airways were similar between the genotypes although PBV eosinophils were lower in the KO mice suggesting that RELM-β may play a role in eosinophil recruitment into the lungs, but once there, their egression into the airways may not be affected. Since matrix metalloproteinase (MMP)-2 promotes eosinophil migration across the epithelium^47^, it would be worth investigating MMP-2 expression in the *Retnlb* null mice. Contrary to a previously identified role for RELM-β as a CD4^+^ T cell chemoattractant^48^, we found more T cells in the airways of RELM-β null mice similar to findings by Liu *et al*. in the bleomycin model^12^. The impact of RELM-β on leukocyte populations in the lungs after allergen exposure may only be minimal, however, it may be important to determine functional responses of each cell type, especially T cells, to RELM-β in order to fully elucidate its role in inflammation.

Cytokines are major contributors to asthma pathology. While we did not measure all mediators in the allergic lungs, a persistent cytokine storm occurred in both genotypes suggesting that RELM-β may not directly regulate these. The absence of RELM-β led to increased expression of *Tlr4* and *Il13*, although there was no major impact on airways or PBV inflammation in *Retnlb*^−/−^ compared to WT. However, increased baseline levels of TNFα, VEGF, and IFNγ in the *Retnlb*^−/−^ mice suggest that RELM-β may be necessary to prevent the host from being in a heightened T_H_1 inflammatory state at homeostasis. As such, it would be of interest to determine if the absence of RELM-β may enhance resistance to intracellular pathogens, similar to its role against worms^9^. Naïve KO mice also had more macrophages as a percentage of total compared to naïve WT mice suggesting a function for RELM-β in immune homeostasis in the lungs. Related molecule, RELM-α, is most notably known for its function as a mediator of T_H_2 responses including allergic asthma^49^. Interestingly, *Retnla* expression was significantly lower in the KO mice compared to WT controls suggesting that RELM-α may not be performing a compensatory role in this model. It is possible that the differences observed between the genotypes were due to the low expression of RELM-α thereby essentially removing both RELMs from immunoregulation during allergen exposure in the KO animals. Alternatively, our data may indicate that RELM-β does not play a direct role in regulating inflammation in response to allergen provocation.

Mucus hypersecretion through GC hyperplasia and metaplasia after allergen exposure can alter lung physiology by slowing ciliary beat and narrowing the airway lumen. Similar to inflammation, GC metaplasia is a feature that is effectively recapitulated in our SAFS model^19,50^. A previous report demonstrated that intranasal administration of *A. fumigatus* antigens to *Retnlb*^−/−^ mice for three weeks caused a ~20% reduction in GCs^11^. Here, we report the contradictory finding that KO mice had increased bronchial GCs and mucin gene expression in response to *A. fumigatus* allergen exposure suggesting that RELM-β may negatively regulate mucus production in the lung during allergy. These data also indicate that *Retnlb*^−/−^ mice may have a different mucin composition, which in turn can have a physiologic impact especially when taken together with the lack of *SurfA* expression. Surfactants are important regulators of fluid balance in the lungs^37^, while mucins can provide nutrients to sustain bacterial colonization^51^. Since asthmatics are considered to be at high risk for bacterial infections^52^, it may be that dysregulated surfactant and mucus in airways during asthma exacerbations can increase susceptibility to subsequent bacterial infections. Gut colonization of germ-free mice leads to an increase in RELM-β secretion in the gut^21^, suggesting that RELM-β is dynamically regulated by host-pathogen interactions. Antimicrobial peptides such as CAMP may be of extreme importance in such instances, and the hyperexpression of *Camp* in *Retnlb*^−/−^ mouse lungs may indicate heightened antimicrobial defences as CAMP is both antibacterial^53^ and antiviral^41^. Additionally, RELM-β is considered a bactericidal agent that controls microbial populations^26^. Since asthmatics have elevated RELM-β^16^ and mucus^54^, investigating the role of RELM-β as a regulator of mucin composition and antimicrobial defences in the lung is of interest especially from the perspective of cause and effect relationship between the microbiome and asthma.

Airway wall remodelling events, including subepithelial fibrosis and smooth muscle cell hyperplasia, can occur in chronic lung diseases such as asthma and chronic obstructive pulmonary disease. The inability to recapitulate this clinical feature is often a criticism of mouse models^55^. Our SAFS model however, results in robust remodelling that continues for months after the fungal challenge^19,56^ allowing us to investigate this phenomenon. Inflammation, subepithelial fibrosis, and mediators such as IL-13 all lead to AHR and we show that the absence of RELM-β results in increased subepithelial fibrosis, AHR, and *Il13* expression. Interestingly, PBV smooth muscle hyperplasia was equivalent between WT and RELM-β null mice (data not shown) indicating that sustained AHR in KO mice may not be due to smooth muscle cell hyperplasia. Whilst IL-13 has been shown to induce RELM-β production^32^, a possible feedback regulation on IL-13 by RELM-β has not been previously demonstrated. Since IL-13 can influence airway inflammation, hyperresponsiveness, fibrosis, and fibroblast functions^57–59^, these characteristics may have been heightened due to *Il13* overexpression in *Retnlb*^−/−^ mice, thereby suggesting a novel function for RELM-β as a negative regulator of IL-13 in asthma.

Collagen is an important extracellular matrix component, that can be detrimental to lungs when dysregulated. We found that PBV collagen deposition was highly elevated in the absence of RELM-β, even at baseline, suggesting that RELM-β may be anti-fibrogenic thereby contradicting several other studies^11,12,16^. While others have noted that RELM-β increases mito- and moto-genic properties^11^ and collagen production^13^ in fibroblasts, our findings suggest that although RELM-β promotes primary lung fibroblast growth, it is not required. Therefore, altered collagen in the lungs may not be due to aberrant fibroblast growth in *Retnlb* null mice. These findings support another study that demonstrated a protective role for RELM-β as an inhibitor non-alcoholic steatohepatitis^60^. We previously demonstrated that the nature of the allergen and the route of exposure causes dynamic changes in asthma pathogenesis^19^. Additionally, the function of RELM-β in the gut may be through dynamic feedback regulation with the microbiome^25,26^. Therefore, it is possible that differences in background strains of founders and the variations in models, together with housing conditions and their possible influence on the microbiome, may also have contributed to differences between our data and that of others.

While the expression and functions of RELM-β may be dependent on a variety of factors, including the immune system and microbiome, elucidating its role in lung and gut diseases during various insults is important to distinguish positive/negative feedback mechanisms that may be beneficial to identify targets for therapy. As the incidence of asthma increases worldwide, understanding the function of immunomodulators in different endotypes of asthma is important to identify novel approaches to personalized therapeutics. Herein, we show that resistin family member, RELM-β, regulates immune responses to allergens and propose that it inhibits the development of chronic features of allergic asthma.

## Materials and Methods

### Ethics Statement

All animal work described herein were performed in strict accordance with protocols approved by the Institutional Animal Care and Use Committee (IACUC, approval number 15-003.0) at the University of Tennessee Health Science Center in Memphis.

### Mouse Model of SAFS

Animals were maintained in micro-isolator cages with alpha-dri bedding and *ad libitum* access to food and water, and housed in a temperature and humidity controlled room with purified air at a 12 hour light-dark cycles. Mice with a targeted deletion in *Retnlb*^23^ backcrossed to C57BL/6 strain were received as a kind gift from Drs. Gary Wu and Angela Haczku. C57BL/6 mice from Jackson Laboratories (Bar Harbor, ME) were used as wild-type (WT) controls. Seven-week old mice of both genders were used as we determined no gender-based differences in the development of SAFS in pilot studies.

Allergen sensitization and challenge were performed as previously described in detail^19,56,61^. Briefly, *A. fumigatus* extract (Greer Labs, Lenoir, NC) -sensitized mice were exposed via inhalation to live unmanipulated conidia liberated from an 8-day old *A. fumigatus* culture (NIH strain 5233, ATCC, Manassas, VA) for 10 minutes, rested for two weeks, and repeated. Untreated mice were used as naïve controls that were euthanized at the equivalence of week 8 in the treated mice. Tissue were collected at predetermined time points after the second fungal inhalation challenge to capture the early and late phases of the allergic response. The timeline of the treatments is schematically represented in Fig. 1.

### AHR Measurement

Anesthetized mice were intubated with an 18-g metal catheter, attached to a computer-controlled small animal ventilator (flexiVent FX1, SCIREQ, Quebec, Canada) and a methacholine dose-response curve was performed as previously described^62^. Peak values for each dose was used to calculate the mean and standard deviation for independent doses at each time point.

### Tissue Harvest and Processing

Animals were euthanized by CO_2_ asphyxiation per approved protocols and BAL was performed. Lavage contents were centrifuged at 1,500 × *g* for 10 minutes to separate the fluid from cells. The BAL fluid was stored at −80 °C until use, while the cells were stained for flow cytometric analyses as described below.

As we have not noted lobular discrepancies in inflammation, GC metaplasia, and airway wall remodelling events in this natural inhalation-induced model of allergic asthma during time when the model was developed and characterized, we divided the lungs for various analyses. Cardiac, middle, and lower half of the proximal right lung lobe were snap frozen in liquid nitrogen, and stored at −80 °C for RNA analyses. The remaining half of the proximal right lobe and the distal lobe were collected for protein analyses. Left lung lobes were inflated *ex vivo* with 10% normal buffered formalin for histology. Pooled blood in the thoracic cavity was centrifuged at 12,000 × *g* for 10 minutes to separate serum which was stored at −80 °C until use.

### Flow Cytometric Analyses

Following red blood cell lysis, BAL cells were incubated in human gamma globulin for 30 min and stained with fluorescently-tagged antibodies for 30 minutes on ice, Mac-3-biotin was probed for using streptavidin-BV605, and cells were subsequently fixed with 1 × stabilizing fixative (BD Biosciences, San Jose, CA). Data from filtered fixed cells were acquired using a LSR Fortessa (BD) cytometer. The following antibodies (clones in parentheses) were purchased from BD Biosciences and used at 1:50 dilution unless stated otherwise: CD8α-FITC (53–6.7), CD19-PerCP/Cy5.5 (1D3), Ly6G-V450 (1A8), CD193 (CCR3)-Alexa Fluor 647 (83103), CD4-Alexa Fluor 700 (RM4-5), CD3ε-PE/Cy7 (145-2C11), NK1.1-APC/Cy7 (PK136), Siglec-F-PE/CF594 (E50-2440), CD107b (Mac-3)-biotin (M3/84, BioLegend, 1:500), Streptavidin-BV 605 (BioLegend, 1:200). Matched isotype antibodies were used at the same concentrations. FCS files were analysed using FlowJo v10.3 (Flowjo, LLC, Ashland, OR) and the Supplemental Figure shows the gating strategy.

### Histological Analyses

Left lungs were sectioned at 4 µm and glass slide affixed sections were stained with haematoxylin and eosin, periodic acid Schiff’s, and Masson’s trichrome stains for analyses of inflammation, GCs, and collagen deposition respectively. All slides were scored by an investigator blinded to the genotypes and study time points.

Ten equivalent areas at 100x magnification along the large airways were scored in one lung section for each mouse in the group. A score of ‘0’ indicates no inflammatory foci in H&E while a score of ‘3’ indicates the most inflammation observed around blood vessels and airways.

Eosinophils in inflammatory foci around the large airways and blood vessels were counted in each section based on the nuclear morphometry and pink staining in the cytoplasm at 1000x magnification. Fifty fields were counted in each sample. GCs were enumerated along 100 µm of large bronchi and represented as the percentage of epithelia lining the airway.

The thickness of trichrome stained collagen fibrils from the basement membrane of the bronchial epithelia toward the parenchyma was measured in ten randomly selected airways in each lung using a calibrated Nikon microscope with NIS-Elements software v4.2. The average in ten fields were calculated for each mouse lung and the mean and standard error of the mean were calculated and reported for each group.

### Quantification of Antibodies

IgA and IgG_1_ (Bethyl Labs, Montgomery, TX) and IgE (BD Biosciences) were quantified in serum and BAL fluid by ELISA. Serum samples were diluted at 1:5000 for IgA and IgG_1_, and 1:500 for IgE while BAL fluid samples were diluted at 1:100 for IgA and IgG_1_, and used neat for IgE. *A. fumigatus*-specific titres of total Ig, IgA, IgG_1_, and IgE in serum and BAL fluid were measured as previously described^63,64^.

### Quantitative real-time PCR

RNA in right lung lobes were extracted with Trizol reagent (Invitrogen, Carlsbad, CA), and 1 µg of RNA was used to generate cDNA with iSCRIPT™ reagents (BioRad, Hercules, CA). Diluted cDNA was used with RNA-specific Quantitect primer sets for *Hprt-1*, *Muc5ac*, *Muc5b*, *Retnla*, *Camp*, *SurfA*, *Il13*, *Tlr2*, and *Tlr4*, with SYBR® Green master mix (all from Qiagen, Hilden, Germany) to determine changes in gene expression using the ABI 7500 (Applied Biosystems, Foster City, CA). Gene expression changes were calculated with the 2^−ΔΔCt^ method relative to *Hprt-1* housekeeping gene and standardized to naïve controls.

### Multiplex Cytokine Assay

Lung lobes were homogenized in 1 mL of Roche cOmplete Protease Inhibitor (Basel, Switzerland) and centrifuged to remove debris. Supernatants were diluted two-fold and quantified using the R&D Systems’ (Minneapolis, MN) Mouse Magnetic Luminex Assays with a Luminex MAGPIX running xPONENT 4.2 software.

### Primary Fibroblast Wound Healing Assay

Fibroblasts were isolated from lungs of WT and *Retnlb*^−/−^ mice following a standard protocol^65^. Cultured fibroblasts were seeded in 24-well plates at 4 × 10^5^ cells/mL and incubated for 24 hrs. Confluent monolayers were scratched with sterile pipet tips, and wounded cells were incubated in media containing 5% of FBS with/without 10 ng/mL of recombinant murine RELM-β (PeproTech, NJ). Cultures were photographed at 20× magnification with an EVOS cell imaging system (ThermoFisher, Waltham, MA) and wound area measured at different time intervals with ImageJ software v1.48.

### Statistical Analyses

Each genotype consisted of 4–7 mice at each time point. The entire study was repeated independently for reproducibility. Data were analysed by two-way ANOVA with Dunnett’s, Sidak’s, or Tukey’s multiple comparisons tests as appropriate using GraphPad Prism software v6.01 (La Jolla, CA) as noted in each Figure Legend.

### Data availability

The datasets generated during and/or analysed during the current study are available from the corresponding author on reasonable request.

## Electronic supplementary material

Supplemental Data
