# Supplementary material for: Chronic features of allergic asthma are enhanced in the absence of resistin-like molecule-beta
Source: Sci Rep. 2018 May 4;8:7061. doi: 10.1038/s41598-018-25321-y (PMC5935686; doi:10.1038/s41598-018-25321-y)
Supplement: Supplementary file 1 — Supplemental Data [file 41598_2018_25321_MOESM1_ESM.docx]

**Title Page for Supplemental Data**

**Title**: Chronic features of allergic asthma are enhanced in the absence of resistin-like molecule-beta

**Authors:** Kim S. LeMessurier,^1,2^ Maneesha Palipane,^1,2^ Meenakshi Tiwary,^1,2^ Brian Gavin,^1,2^, and Amali E. Samarasinghe^1,2,*^

**Affiliations**: ^1^Department of Paediatrics, University of Tennessee Health Science Center, Memphis, TN 38103; ^2^Children’s Foundation Research Institute, Memphis, TN 38103.
